# Supplementary material for: Spatial localization ability of planarians identified through a light maze paradigm
Source: PLoS One. 2023 Jul 19;18(7):e0288118. doi: 10.1371/journal.pone.0288118 (PMC10355441; doi:10.1371/journal.pone.0288118)
Supplement: Attachment — Submitted filename: Response to reviewers.docx [file pone.0288118.s002.docx]

Major concerns:

Is it correct that successful worms follow the same trajectory as the previous successful trials? Why not calculating the trace accuracy using the data? In fact, you show only two figures regarding the data analysis. Maybe too little analysis? Linked to this, is there a difference regarding the speed of worms between the first trial and the other trials? I wonder the speed of worms is affected by the learning accuracy.

We thank the reviewer for pointing out these issues. However, after trying some software, we could not find an effective tracing software that can accurately trace the worms. Because when the worm climbs on the sidewall of the maze, the software loses the target. This might also happen when a worm wriggle and causes its body to change shape. Therefore, we chose to draw some routes to prove that the worms could somehow get to the dark chamber. Also, by roughly estimating the worm’s trace, we did not find a general rule for how the worms got to the dark chamber. Even so, our video can be provided and used for analysis by other researchers. We also counted the speed of the worms and found that worms are getting slower after the first trial, which leads to a respectively longer latency for worm to get to the dark chamber. Data analysis of the speed of the worms is now added in the result part.

I could not find any statistical explanations regarding the data in Results section. Linked to this, why not adding some statistical analysis to results shown in fig 2B? Are there any statistical differences regarding the ratio of worms reached criterion between trial 1 and the other trials?

We thank the reviewer for pointing out this issue. We apologize for omitting statistical analysis. Statistical analysis of the original data is now added to the result part.

I wonder what kind of spatial information worms rely on. What happens if some geometry arrangement in the light maze is modulated after several trials? Also, you may as well consider the rearrangement of the position of the dark chamber after several trials. Could you expand discussion regarding this?

We thank the reviewer for this suggestion. Due to technical restrictions on our route tracing method, we are still working on this question. The discussion part is expanded regarding to the suggestion.

Minor comments:

Training procedures: The training procedures is also written in the first paragraph of Results section. I recommend you that you should move it to Method section. In addition, I found that you said in the “Training Procedure” of Method section that 24 worms were used in the training. However, in Result section, you said that 35 worms were totally used. Which is correct?

We thank the reviewer for pointing out this issue. The number of worms is now corrected.

Fig1B: Can you make fig1B a little bit larger? In addition, the two boxes on the far left appear to be different in color from the others. Why?

We thank the reviewer for pointing out this issue. Fig1B is now larger. The photosensitive resin material tends to change its color to a little yellow when exposed under light. However, since planarians do not have visual abilities, we don’t think that this factor might affect the result.

fig2C-F: Although you present some trajectories, I would like to see some movies.

We thank the reviewer for this suggestion. The movie can be provided.

Reviewer #2: The paper suggests a new light-maze system for studying spatial orientation in Planarians.

The idea and the system are interesting and has merits. The paper itself however have some serious flows. The first and most important is that the entire statistics section is completely missing.

There is indeed a part titled "statistics", but it contains no relevant information whatsoever. The results parts follows the same theme – there is a paragraph titled "results" but in actuality it is just an extension of the "Methods" part. Also, because the paper contains no statistical tests, there are no real results reported anywhere in it.

These two issues alone should disqualify the paper, to my opinion.

However, because of the somewhat novel and interesting system – I think it is worth publishing after some major revisions. Mainly: (a) adding the full statistics, (b) rewriting the "Results" and "Methods", and (c) measuring light levels on the maze surface (there is no need to re-run the entire experiment – only arrange the set up as it was and measure light levels across the maze, maybe at the beginning, middle and just above the dark solution patch).

There are also various other issues in the paper which I will list below.

We thank the reviewer for pointing out these issues. The statistics were added and the relevant parts were rewritten as suggested.

Introduction.

Line 15: Please elaborate more or at least give an example regarding the differences between the “ability to discriminate directions” and “complex localization ability”.

We thank the reviewer for the suggestion. The relevant part is now elaborated more as suggested.

Line 15-24: This part deals with the Morris water maze and the light-based system the authors offer. The authors’ suggestion and design are solid, but they are in fact a combination of the Morris water maze and the Barnes light maze. I therefore feel like the latter should be at least mentioned here.

We thank the reviewer for the suggestion. The Barnes light maze is now mentioned in the text.

Line 24-25: (“The planarians’ primitive eyes can only sense the light rather than imaging; thus, they cannot visualize the dark chamber”. I am not sure ‘visualize’ is the correct word here and would have chosen a slightly different phrasing.

We thank the reviewer for pointing out this issue. The sentence is now rephrased as “The dark chamber is not visible to the worms.”

Line 26: The text jumps to introduce “trial 4” without describing first the experimental design or mentioning previous trials or their purpose. I would (a) consider moving this entire paragraph to “Methods” (b) change “trail 4” to “test phase” or something similar along these lines.

We thank the reviewer for pointing out this issue. The phrase “trail 4” is changed to “test phase”.

Line 31: “This finding proves that the planarian worms have spatial localization ability, which is not yet discovered”. One cannot claim to report something which is not yet discovered. I would rather write something like “these are the first finding suggesting that planarian worms have spatial localization abilities”.

We thank the reviewer for pointing out this issue. The sentence is rephrased as suggested.

Lines 34-35: (“Also, since the planarians’ nervous system has simple structures, it can

provide a model for spatial learning studies.”). This sentence is true and it makes sense. However, it remains at the statement level if there is no follow up, elaboration or a suggestion for further research. Similar statements repeats through the paper, but still without any concrete suggestions.

We agree. In fact, currently, there is little research about planarian behavior, nor did we find any neural circuit studies of planarians, possibly due to the difficulty of gene editing in planarians. Therefore, we could not currently give concrete suggestions and we hope that further studies can solve this problem to help establish this field.

Materials and Methods.

Line 3-4: First, past form is preferable here (worms were instead of worms are). Second, I feel like a short explanation of what exactly “Montjuic Water” are is due here. I for once don’t know this term and reference 13 is locked behind a pay-wall. Also, if this is indeed a standard procedure in planarian rearing I would like to see some references to this method: for example “Worms were maintained in Montjuic Water in the dark (like in XX et-al. 2019, YY & ZZ 2022) etc….”

Finally, food amounts and preparation methods are missing: How much chicken liver? In what form? Was it whole? Crushed? Minced? Was it mixed in the water? Was it removed the next day?

We thank the reviewer for pointing out this issue. We actually used protocol from M. Shane Merryman et al. 2018 for reference and this case is now illustrated in this part.

Experimental Setup.

Line 2: Why are the maze dimensions not mentioned in the text? Please describe the maze and mention them before you are referring me to the Figures (although they should be mentioned there too)

We thank the reviewer for pointing out this issue. The maze dimensions are now added to the text.

Line 3: Extra ‘r’ in ‘were’ (here the paragraph is back to past tense, which is good). Also, why was photosensitive resin used for the maze? It is probably nothing and I am sure the authors have a good explanation and they thought this out. But since this paper deal with light mazes and it effect on small creatures I would have like to see this issue somehow been addressed. It would be reassuring to the reader to explain why it is not a problem.

We thank the reviewer for pointing out this issue. The explanation of this issue is now added to the discussion part.

Line 6: (“The inner side of the mazes is coated…”). We are back to present tense, please don’t jump from tense to tense, at least not within the same paragraph.

We thank the reviewer for pointing out this issue. The tense is now corrected.

Line 10-15: This discusses the lightning of the maze. In light maze experiments it is common to measure and report the illumination level (in Lux), rather than state the specifications of the lightning apparatus. If specifications already appear, I would also like to see the makers/brand of the Led tube reported – This could help a lot in recreating the experiment.

In light of the the results mentioned in the Discussion – It would be a good idea to re-set the experiment (no animals or water needed) and to measure and report the light level at least at the beginning and end points of each maze. Otherwise the authors cannot claim the worms relied only on distal cues to solve the maze.

We thank the reviewer for pointing out this issue. The lighting conditions is now added in the method part.

Training procedure:

This entire part is very unclear and inconsistent. I feel it should be re-written completely and therefore there are no line numbers stated here but only general remarks.

Some unclear and troubling points:

We thank the reviewer for pointing out the issues below. The entire part is now rewritten and clearly illustrated the issues below.

3. this sections starts with: “We used 6 mazes to train 24 worms in the first 3 trials”.

Were the worms trained individually or in groups of 4? Did water or mazes were switched between training runs? (Something like that was mentioned before – but it is not mentioned here).

2. Next the text states: “total of 6 trials in 6 days” – isn’t this come up to a total of 34 trials (6 treatments X 4 trials equals 24 trials). What is the reason for this discrepancy?

3. “To control worms’ satiety, they were fed at 10:00 a.m. on day 1 and day 4”

When were the worm fed? On Sunday and Thursday as mentioned before? A day before training? Were Sunday and Thursday both were a day before training? This is very unclear.

Also – Why is important to control satiety when there is no food reward in the maze and the initiative to solving it is light avoidance? If it is only for standardization, say so. If there is another reason – mention it.

Satiety control is for standardization and is illustrated in the discussion part.

4. “Then the water in the mazes is changed (add to 15 mL) and the mazes were set up as described above.” – Well, (a) nothing is described above, (b) were the water changed or were water added to fill a certain minimum? Also, why?

Water is changed. Now, the water amount added to the maze is illustrated in the Experimental Setup part.

5. While re-writing this section, it might be useful to add an explaining diagram describing trials, times, number of worms and the entire experiment process.

A diagram is now added.

Statistical Analysis:

This is the entire statistical analysis section: “Statistical Analysis All data were analyzed using PRISM (GraphPad Prism 9.0.0(121)). Routes are drawn by hand with Photoleap (2.13).”

Need I say more? Please add Statistics…

Also, please explain the process of drawing the routes, as I understand it – Photoleap is a drawing software, not a movement tracking one.

We thank the reviewer for pointing out this issue. We apologize for omitting statistical analysis. Statistical analysis of the original data is now added to the result part.

Results:

This part should also be re-written. In reality it is not a result section but a continuation of the “Methods” part. This is probably somewhat due to the fact that since the statistical tests are missing, there is nothing to report.

This part should contain only the results of each important step of the experiments (including the test-names, the correct statistic for each test, the number of repetitions/iterations and the numbers of animals used in each such tests).

This entire sections should be re-written and moved to the methods, so here too – I will address some issues in general. Some of these are important and alarming.

We thank the reviewer for pointing out these issues. The entire section is now rewritten, and problems described below were all solved.

3. The numbers of worms and trials here are different from what is described earlier (in the methods section).

Why were 35 worms here and either 24 or 34 in previous part?

The executions times are also different from what is mentioned in the methods (although this might be the result of an am/pm typo). If this is the results section – how come a different number of worms was used? If this is actually a continuation of the Methods section – why is it under Results?

The number of worms is now corrected. The entire section is now rewritten.

2. Worms’ satiety is mentioned again here – the question remains, why is it important in the framework of a light maze?

Satiety control is for standardization and is illustrated in the discussion part.

3. “During the training process, only **partial worms** showed learning and got to the dark chamber **fast** in each trial.” (a) I assume “partial worms” means “some worms”? Please rephrase. (b) What does fast mean here? Is it important? (explain why), is it trivial? (omit it).

Is “fast” on a scale? Is it only binary fail/success? More importantly – are Fast/Slow definitions meaningful within your experimental design? If so, they need to be defined, measured and reported accordingly.

The entire part is now rewritten and the sentences relevant were rephrased.

Lines 3-4: “In each training trial, a planarian was put in the start point (opposite the dark chamber corner)”. Please add this start point to the figures. Do this not only in the hand drawn route figures (in which the starting point slightly differs – but also in the relevant places in Figure 1.

The start point is now illustrated in the text.

Line 14: “If a worm stopped moving outside the dark chamber for more than 20 mins, its data from that single trial is excluded” – What does that mean? I think it means that worms that didn’t move for more than 20 min. were omitted from the statistics. But I can’t be sure. Also, because you don’t report the statistics, it is hard to understand if or how meaningful it is. Please explain/rephrase and include all relevant information (including the number of cases where this happened). Also elaborate about your “learning criterion” while at is. When were 40min. used and where did you use 20 min.?

The relevant part is now rephrased and clearly illustrated.

Line 16-17: “From trial 3 to trial 6, there were 10-12 worms that showed learning effects in each trial (Fig2.B). This part starts to resemble “results”. Please phrase it more clearly. Also you have to mention (clearly) somewhere what is your exact “learning criterion” (Is it “worm got to the shade in under 20min.? something else? Don’t leave me guessing) and add it to the graph/figure. Also add the statistical test results (is 7 in trial 2really statistically different from 10 in trial 4? Should I guess here too?).

The relevant part is now rephrased and clearly illustrated.

Lines 17-18: “Comparing trial 4 to trial 2, 3, 5 and 6, there is no significant difference, showing that the worms might not use chemical cues in this case (Fig2.A)”. Here you are jumping to a different graph showing something different than the one two lines ago, yet you are still speaking about trial numbers only (rather than training/test runs or other more informative terms).

This is very confusing and should be corrected. More important, you are claiming there are significant differences here but do not bother to back this statement up: What test did you use? What were the results? Where are the statistical values?

The relevant part is now rephrased and clearly illustrated.

Discussion:

Lines 3-4: Why are these lines relevant here? It has nothing to do with either the lines preceding or following it.

We thank the reviewer for pointing out this issue. The sentence redundant and is now deleted.

Lines 6-8: examples needed here - also, are you sure this belongs in the discussion and not in the introduction?

We thank the reviewer for the suggestion. However, this point is too complicated to explain in this section and is well illustrated in the reference 15. Thus, we decide not to give an example here. And this part is relevant to the next paragraph that we are going to discuss, so we think that this belongs in the discussion part.

Lines 10-14: You are comparing between planarians and mice here. While It is true that one animal have a simpler brain than the other, just stating this fact does not make it a "great investigation point" – please back this statement up by either explaining why is it such a great opportunity and/or at least suggest a theoretical way to test this.

We thank the reviewer for the suggestion. However, there is currently zero investigation on behavior and neural circuit of the planarian worm. Thus, could not offer a theoretical way to test this. Anyway, we rephrased the sentences to make it sound reasonable.

Lines 14-16: the lines state "As in our paradigm, we applied the light maze paradigm to identify the spatial localization ability of the planarians, which can be regarded as true learning and memory."

First, the words "As in our paradigm" does not connect to the previous sentence. Second, please explain what do you mean by "true learning and memory" (are there false learning and memory within your paradigm? Do you mean to differ it from other kind of learning? If so – which? And why?). Otherwise just omit this part.

We thank the reviewer for pointing out this issue. The sentence is now rephrased.

Line 24-25: "The results demonstrated that…"

1. Normally this is how you should have started the discussion.

Here however there are no results. What appears under "Results" is actually a continuation of the methods section and the Statistics and Results sections are completely missing.

2. "A worm might show learning effects in some trials but not in other trials." – While this can happen, you can't just wave this aside without addressing the issue. You need to state why do you think it happened, how often, what does it mean, and if it is a problem or not. Although some explanations follow, they are confused, not sufficient and does not address some of the issues I just mentioned. For example, you cannot just say "the planarians navigation system is primitive and that is why they can solve some tasks and not others". Please explain at least what tasks you are referring to (not to mention what sets apart the unsolved ones). Also, from the initial phrasing it follows that worms also differed in learning results within the same task. Please rephrase to address these issues.

We thank the reviewer for pointing out these issues. The sentences are now rephrased.

Lines 34-35: The authors suggests the "worms used the light intensity gradient combined with the geometric information of the maze to navigate to the dark chamber." – This is another reason why it is important to measure the light gradient across the experiment arena. As already mentioned above – this is something I like to see done before submitting this paper again. Also, how exactly was the geometric of the maze used by the worms?

Finally, as was mentioned by the authors earlier in the article – the worms can detect also vibration, and magnetic and electric fields, how did they account for, or controlled these variables? (i.e - no talking in the experiment chamber, isolating electric devices etc...)

Line 36: "we identified the spatial localization ability of planarians by presenting a new paradigm using the light maze we designed." – No you did not, you cannot make this claim without presenting the statistics supporting it. Also, as mentioned above - this paradigm is far from being new. If anything, you applied an existing paradigm to a new model animal.

We thank the reviewer for pointing out this issue. The word “new” is now deleted.

Lines 38-39: "In this work, planarian worms were found to use distal cues instead of proximal cues to navigate to the dark chamber. I am sorry, but I have to ask again – can you really say that? The authors themselves just stated light gradient as a possible explanation for their findings, and a few other factors (electric and magnetic gradients from the led, earth and/or other lab equipment) could also have influence the results. Please measure the light gradient and also rephrase this part.

We thank the reviewer for pointing out this issue. The part is rephrased.

Lines 40-41: "Identifying the spatial localization ability of such a primitive invertebrate might provide insights into the evolution of spatial learning". Once again, this stays at the statement level. Even if this is true, please elaborate how this provide insights, what kind of insights do you think it will provide, what do you suggest as a direction for future research, where do you think this should go next – things like that..

We thank the reviewer for the suggestion. However, there is currently zero investigation on behavior and neural circuit of the planarian worm. Thus, we feel sorry that we could not expand this topic.

Figures: Figure 2C,D,E,F – These suppose to represent the learned routes of the worms. However, as the text indicates they were drawn by hand using Photoleap. Since Photoleap is not a movement tracking software but rather a 'freehand' drawing program, I would like to see more information regarding the methods by-which the worms movement was translated from the test arena to the final figures. Also, why is the start point in each of them slightly different?

We thank the reviewer for pointing out these issues. However, after trying some software, we could not find an effective tracing software that can accurately trace the worms. Because when the worm climbs on the sidewall of the maze, the software loses the target. This might also happen when a worm wriggle and causes its body to change shape. Therefore, we chose to draw some routes to prove that the worms could somehow get to the dark chamber. So, all the routes were drawn freehand through watching the video with Photoleap. The start point in each of them is slightly different because because the worm need to sink to the bottom, and this is when we started drawing.
