# Supplementary material for: Spatial localization ability of planarians identified through a light maze paradigm
Source: PLoS One. 2023 Jul 19;18(7):e0288118. doi: 10.1371/journal.pone.0288118 (PMC10355441; doi:10.1371/journal.pone.0288118)
Supplement: Attachment — Submitted filename: Response to reviewers.docx [file pone.0288118.s003.docx]

6. Review Comments to the Author

Please use the space provided to explain your answers to the questions above. You may also include additional comments for the author, including concerns about dual publication, research ethics, or publication ethics. (Please upload your review as an attachment if it exceeds 20,000 characters)

Reviewer #1: The authors have satisfactorily addressed most of my concerns.
I wonder why worm speeds in trial 1 are significantly higher than other trials.
You may or may not discuss this.

We thank the reviewer for pointing out this issue. When worms encounter an unfamiliar environment, their speed becomes high in order to get familiar with the environment as soon as possible, as worm speed in trial 1 is significantly higher than worm speed in other trial, and worms’ speed gradually decreases trials after trials. Interestingly, when worms are transferred from an old maze to a new maze (maze pattern is the same), their speed slightly increases, as worms’ speed in trial 4 are slightly higher than trial 2, 3, 5 and 6.

Reviewer #2: Lines 17-21: These lines suggest claims that the worms can navigate to a formerly recognized place from a start point. For these claims to hold water they need to meet at least one of two conditions: (1) did routes became shorter between runs? (There is no evidence in the text for that), (2) did solution times became shorter between runs (according to the authors answer to the first reviewer, it seems they actually got longer). Unless at least one of this conditions is satisfied, how do we know that the worms do not just randomly swim until they find the save dark patch?

We thank the reviewer for pointing out this issue. In figure 1.A, we can see that solution times became shorter between runs. In trial 1, the solution time is significantly higher than that of trial 4 (worms were transferred to a new maze). The short solution time means that learned worms can navigate to the dark chamber in a new maze. In Figure 2.D-G, we can see that routes became shorter between trial 1 and trial 4. However, we could not evaluate the exact route distance in each trial due to technical difficulties as demonstrated in our former response. The relevant descriptions are now added to the result part.

Lines 50-51: I would rephrase these lines or completely omit them. (I think I can guess what the general meaning here is, but it is badly written and confusing and I am not sure how much it contributes to the overall understanding of the design).

We thank the reviewer for pointing out this issue. The sentence is now rephrased.

Lines 84-85: Here I would have liked to see some reference to the photosensitive resin used. I asked for this matter (why use a photosensitive resin in such an experiment design when photosensitivity is key) to be clarified during the previous revision – and I am asking for this again now.

We thank the reviewer for pointing out this issue. There are mainly two types of 3D printing techniques currently, fused deposition modeling (FDM) and the stereolithography (SLA). The objects produced by FDM are porous and water leaking, which made us not to consider this technique in making the main body of the maze. Also, the SLA 3D printing method which uses photosensitive resin as raw material behaves better in 3D-printing speed and resolution. The photosensitivity of the resin means that during 3D fabrication, the liquid resin cures after exposed to the UV light. After curing, the resin becomes stable, solid and can no longer change its physical properties under any kind of light. Therefore, the photosensitivity of the resin had no relevance to the light in our maze experiment. The reference and the illustration above are now added to the Materials and Methods part of the manuscript.

.Line 107: please add the missing space between "changed' and "and".

We thank the reviewer for pointing out this issue. The missing space is now added.

Lines 117-119: The authors state "Light intensity variance along Wall a is less than 5 Lux, which can be neglected" - Reference is certainly needed here. Also light level should be measured not only from the wall of each maze but also in points between the maze 'entry' and maze 'solution'. From figure 1D it seems quite likely that there is might be some brightness gradient there.

We thank the reviewer for pointing out this issue. Actually, what we wanted to express is that in a typical maze, the light intensity variance (LIV) along wall b is more than 100 Lux, which is much larger than its LIV along wall a, which is less than 5 Lux. So, if the worms relied on the LIV to navigate, we think that they would rely more on the LIV along wall b than wall a. To avoid misunderstanding, we have now deleted the phrase “which can be neglected”. Based on illustration above, the light level between maze 'entry' and maze 'solution' should be ±5 Lux from our measurement in figure1.D, Based on this, we think it is no need to measure light intensity from everywhere in the maze.

Line 123: This is the first time the term "latency" appears in the text. I do not understand what it means here (or for that matter, when you use it again and again in the following parts of the text). Please define this terms clearly as it seems important to your claims. I would have preferred for it to be discusses or defined in the materials and methods section or in experiment design – but it doesn't really matter as long as you define it clearly the first time it appears in the text.

We thank the reviewer for pointing out this issue. The relevant definition is added from line 71-75 in the introduction part.

General remarks for the "statistics" chapter:
This is poorly written and does encourage trust in your results. Please phrase your research questions (preferably at the end of the introduction part), then report the statistical tests you chose to test these questions and why. This part should also include the number of repetitions and of worms in each state (not only of the ones used in the tests, but also the worms disqualified and the reasons for doing so).

Important: Note that you are mentioning specific tests in the "statistics" section and then report the result of *different* tests in the result section.

We thank the reviewer for pointing out this issue. The research question is now added to the end of the introduction part and illustrations of statistical tests are now added to the statistics chapter.

Lines 142-143: This would be a good place to state the total number (N) of the worm which the authors actually included in the statistics. Also, I do not completely understand what "latency' mean here too – please add an explanation. While we are on this subject – please state clearly not only the N for participating worms, but also for the disqualified ones.

We thank the reviewer for pointing out this issue. The number of worms counted in each trial is now added to the statistics part. The definition of latency is added from line 71-75 in the introduction part.

Lines 145-145, 150-151: I would choose a term other than 'escape' and clearly define it. If you choose to remain with 'escape', please add a definition or an explanation for it (does it mean 'solved the light maze?', 'escaped the arena'? something else?). Also fix the extra dot/period in line 150. Note that in line 207 in the discussion (for example) you are using 'escape' in yet another context.

We thank the reviewer for pointing out this issue. The relevant definition is added from line 71-75 in the introduction part. The extra dot/period in line 150 is now fixed.

Lines 147-157:
a. these lines states: "To test whether this behavior is stable after changing the maze, we continued trial 5 and trial 6. comparing trial 4 to trial 2, 3, 5 and 6, there is no significant difference, showing that the worms might not use chemical cues in this case (One way ANOVA, P>0.1; Fig.2A)". The text does not specify what was compared to what?! What was the comparison criteria? Speed? Number of solutions? What is behind the statement that this comparison shows "that the worms might not use chemical cues in this case"

We thank the reviewer for pointing out this issue. The detailed explanation of the sentence is now added.

b. If there is significant difference between trial 1 and all other trails, indicate it - as you did in 2A. But this is a minor problem. If solving speed declines with trials (as stated in lines 154-157), how can you tell the worms learned? How can you say that there were more "solves" (if that is what 'escapes' means) in advanced trials because of learning and because of random chance (e.g., worms explored the maze for longer times).

We thank the reviewer for pointing out this issue. There is a logical relationship that if the speed declines with trials, it takes more time for the worms escape the maze, which makes it harder for worms to show their learning effects. However, the decline of the speed did not affect the worms’ escape latencies, so it won’t affect the conclusion that worms learn to escape the maze faster after training.

c. I ask again, what exactly is "latency" in your experiment system? Also, why use 241mm. in some cases and actual moving distance in others? You are pooling two different data sets together and then running a single test on the new pool? Why? Please explain this.

We thank the reviewer for pointing out this issue. The definition of latency is added from line 71-75 in the introduction part. 241 mm is the perimeter of the maze. For worms which can quickly get into the dark chamber, its moving distance is usually less 241 mm, so we can only use its actual moving distance to count its speed. For other worms who usually climb along the wall of the maze, it is more accurate to use the maze perimeter to count its speed, so we chose 241 mm to count its speed.

Lines 162-163: Please state how many worms we are talking about (this is already the results section and still we don't know how many worms were used for the statistics). What are the learning effects? As mentioned before – if solution times does not diminish and/or solution routes does not shorten, what is the criteria for learning?

We thank the reviewer for pointing out these issues. These issues can be solved by my responses on other issues above: through definition of latency and illustration of effect of worm speed on latencies.

Line 169: Please add a space between "40" and "min.

We thank the reviewer for pointing out this issue. The space is now added.

Lines 171-174: please fix this entire paragraph: the worms' routes Illustrations starts with 2D and not with 2C as appears in the text. More important issue here is that for some reason, in the advanced trials illustrations the end point of the worms *are not* under the dark chamber (while ending under it in the first trial). It seems like, at least from the illustrations that in opposition to the authors claims – the worms did not learn.

We thank the reviewer for pointing out this issue. The worms' routes Illustrations are now fixed. The end points were outside the dark chamber is because our camera could not catch the worm routes inside the dark chamber. Anyway, all worms that solved the maze were identified that they were in the dark chamber after the test. And we can provide videos to demonstrate this issue.

General remarks for the "Results" chapter:
I would like to see a better written Results part. This should include better definitions of what was measured. (for example: what is 'latency'? what are 'escapes'? how was 'speed' calculated? Why there is no report regarding routes length). Please describe exactly what was measured or tested under each such definition or terms. Do so before or when you are introducing this term for the first time.

We thank the reviewer for pointing out these issues. These issues can be solved by my responses on other issues above: through definition of latency and illustration of effect of worm speed on latencies, and illustration of routes length.

You should also state what exactly was compared to what? Did solution times change between trial 1 and 4? If yes - To what direction? If not – why and what criteria was compared? Why did you choose to compare trial 1 to 4 and not 1 to 6?

We thank the reviewer for pointing out these issues. These issues can be solved by my responses on other issues above: through definition of latency and illustration of effect of worm speed on latencies, and illustration of routes length.

Trial 4 uses newly manufactured mazes to test the worms, in this case, worms cannot use local cues such as chemicals for navigation, so we compare trial 1 to 4 and not 1 to 6. This illustration locates at the first sentence of the result section.

I would also like to see the actual number of animals eventually used in each statistical test as this is very unclear in the current text (something is mentioned under fig.2 but it is unclear). The N should appear also in the manuscript main text next to each relevant test. This is especially true when some of the N values mentioned are a bit too small for comfort).
Eventually, I cannot understand why you are describing statistical tests in the 'statistics' section and report results from completely different tests in the results. Please take care of this issue.

We thank the reviewer for pointing out these issues. We have now added detailed explanation of these issues in the “Statistical Analysis” chapter.

Lines 177-184: These lines belong in the introduction. Please start the discussion with shortly describing your main findings. Then you can move on to explain them in relation to other work in the field or if there is none – their novelty. Either way though - these lines are not relevant here.

We thank the reviewer for pointing out this issue. The discussion part is now started with shortly describing our main findings.

Line 192: It seems like the word 'including' is unnecessary here.

We thank the reviewer for pointing out this issue. The word including is now deleted.

Lines 200-211: these lines also do not belong in the discussion. The process of manufacturing 3d mazes, their toxicity and the material used should appear in the introduction and in the method parts. You can of course stress the novelty and advantages of your design and techniques in the discussion – but not to delve into a deep discussion of materials and method. It belongs elsewhere. Also, please notice that in line 207, you are using 'escape' in yet another context.

We thank the reviewer for pointing out this issue. Related parts are moved to the method part.

Lines 212-214: These are the lines the discussion part should have begun with. However:
a. you are using 'escaped' in yet another context here.

b. It is not clear if the results are neither significant or actually support the authors claims.

c. This brings us back to the results section. Please rewrite it.

We thank the reviewer for pointing out these issues. These issues can be solved by my responses on other issues above: through definition of latency and illustration of effect of worm speed on latencies, and illustration of routes length.

Lines 214-218: What does this mean for the results? How many times did this happen? This should be stated in the results section. Also, what do the authors mean by "worms cannot accurately solve every task"? Did they control for each worm in successive trials and singled out ones that repeatedly could not solve their light maze? Something else? Since there is no report of the proportions of 'solvers' vs. 'non-solvers' the reader can not evaluate the meaning or strength of this statement.

We thank the reviewer for pointing out these issues. We have now added these information in the result part. However, there are only 3 worms in 36 worms that could solve all 5 trials. Other worms can only solve 0-5 trials for some unknown reasons.

Lines 230-234: In these lines the authors claim that "In this work, planarian worms were found to use distal cues instead of proximal" and that "planarians are now the most primitive animal found to have spatial learning ability".
In light of the remarks above, I am yet to be convinced: What were the definitions for proximal and distal in this experimental setup? According to what hypothesis or tests the authors reached these results? These should appear in the manuscripts, ideally at the end of the introduction.

We thank the reviewer for pointing out these issues. However, we did not explore which kind of proximal and distal cues that the worms relied on. Anyway, we listed possible proximal and distal cues in the 3^rd^ paragraph in the introduction part.

General remarks for the discussion:
The discussion also need some rewriting: The entire opening paragraph (lines 177-184) describes the history of the research field and belongs (if at all), in the introduction. The discussion then moves to explain reasons for the results (which should have been briefly summarized in the opening of the discussion). Directly after that the text explains the benefits (and some of the problems) of 3D printing and only on line 212 (35 lines into the discussion) finally some of the results first appear. This is structurally wrong and needs to be fixed.

We thank the reviewer for pointing out these issues. These issues can be solved by my responses on other issues above.

What worries me more, however, is that what described in the 'results' section not necessarily shows that the worms actually learned between sessions: what is the criteria? Learning speed? Total numbers of worms that found the shelter in each trial? Shortening routes? Did the researchers followed specific worms and saw better performance? The results chapter lack clarity and the research hypothesis are not mentioned, therefore it is hard to understand what on what bases the authors' states these results.

We thank the reviewer for pointing out these issues. These issues can be solved by my responses on other issues above: through definition of latency and illustration of effect of worm speed on latencies, and illustration of routes length.
